# Differences in Cancer Death Risk Long After ACS Among Selected Urban and Rural Areas in North Italy: The ABC-7a^†^ Study on Heart Disease

**DOI:** 10.3389/fonc.2021.731249

**Published:** 2021-10-14

**Authors:** Heba T. Mahmoud, Giuseppe Berton, Rocco Cordiano, Rosa Palmieri, Tobia Nardi, Mohammad AK Abdel-Wahab, Fiorella Cavuto

**Affiliations:** ^1^ The ABC Heart Disease Foundation-Organizzazione Non Lucrativa di Utilità Sociale (ONLUS), Conegliano, Italy; ^2^ Department of Cardiology, Conegliano General Hospital, Conegliano, Italy; ^3^ Department of Internal Medicine and Cardiology, Adria General Hospital, Adria, Italy; ^4^ Department of Cardiology, Minia University, Minia, Egypt; ^5^ Department of Cardiology, Bassano del Grappa General Hospital, Bassano del Grappa, Italy

**Keywords:** acute coronary syndrome, cancer death, urban-rural, geographic areas, survival analysis

## Abstract

**Background:**

An increased risk of cancer death has been demonstrated for patients diagnosed with acute coronary syndrome (ACS). We are investigating possible geographic risk disparities.

**Methods:**

This prospective study included 541 ACS patients who were admitted to hospitals and discharged alive in three provinces of Italy’s Veneto region. The patients were classified as residing in urban or rural areas in each province.

**Results:**

With 3 exceptions, all patients completed the 22-year follow-up or were followed until death. Urban (46%) and rural (54%) residents shared most of their baseline demographic and clinical characteristics. Pre-existing malignancy was noted in 15 patients, whereas 106 patients developed cancer during the follow-up period, which represented 6232 person-years. No difference in the cancer death risk was found between the urban and rural areas or between southern and northern provinces (hazard ratio [HR] 1.1; 95% confidence interval [CI] 0.7–1.7; *p* = 0.59 and HR 1.1; 95% CI 0.9–1.4; *p* = 0.29, respectively) according to the unadjusted Cox regression analysis. Geographic areas, however, showed a strong positive interaction, with risk increasing from the urban to rural areas from southern to northern provinces (HR 1.9; 95% CI 1.1–3.0; *p* = 0.01). The fully adjusted Cox regression and Fine-Gray competing risk regression models provided similar results. Interestingly, these results persisted, and even strengthened, after exclusion of the 22 patients who developed malignancy and survived to the end of follow-up. We did not observe an urban/rural difference in non-neoplastic death risk or a significant interaction between the geographic areas.

**Conclusion:**

Our analysis reveals that the cancer death risk among unselected ACS patients in Italy’s Veneto region significantly differs by geography. The northern rural area has the highest risk. These results highlight the importance of implementing a preventive policy based on area-specific knowledge.

## Introduction

Heart disease, especially ischemic heart diseases, and malignancy are considered the major two causes of death worldwide (1, 2). The diseases are linked by inflammation and oxidative stress, which contribute to the development and progression of both. Modifiable risk factors such as tobacco smoking, a sedentary lifestyle, unhealthy diet, and obesity are reported to be major contributors to the pathogenesis of both diseases, possibly reflecting a shared biology (3–7).

An increased risk of malignancy and cancer death was recently reported after acute coronary syndrome (ACS) (4, 8–10) and several epidemiological studies presented the higher risk of incident cancer in patients with cardiovascular diseases, especially CAD.

The SHIP (Sakakibara Health Integrative Profile) cohort study that enrolled a total of 32095 participants with cardiovascular diseases showed that cancer incidence and mortality were >2‐fold higher in patients with atherosclerotic cardiovascular disease (including coronary artery diseases) than in patients with nonatherosclerotic cardiovascular diseases (11).

Moreover, the incidence rates of cancer and death were evaluated in participants with myocardial infarction (MI) from Danish registries which examined 2,871,168 individuals. During follow-up, 122,275 developed an MI and 11,375 subsequently developed cancer (9.3%, IR 19.1/1000 person-years) and 65,225 died (53.3%, IR 106.0/1000 person-years). The study showed that MI was associated with an increased risk of overall cancer compared to the reference population (adjusted IRR 1.14, 95% CI 1.10–1.19) (12).

In another large general cohort study (28,763 participants) with prospective design and 16 years of follow-up, patients who developed MI had a 46% higher hazard ratio of cancer compared to subjects without MI (multivariable-adjusted HR 1.46; 95% CI: 1.21–1.77) (13).

Our group also reported a higher incidence of cancer and neoplastic mortality in patients’ post-MI compared to the general population (4). We have observed that cancer risk in patients long after ACS significantly differs based on the geographic distribution in six urban and rural areas in the Veneto region in northern Italy, with the highest observed risk in northern rural areas (14).

In this study of the same region, we aimed to investigate the existence of differences in cancer mortality among unselected patients who survived the index hospitalization for ACS and were followed for 22 years.

## Methods

### Patients

The ABC Study on Heart Disease (www.abcheartdiseasestudy.org/en/) is an on-going prospective study. It was designed to investigate an unbiased ACS patient population. Specifically, the study includes all consecutive Caucasian patients admitted between June 1995 and January 1998 to intensive care units at three general hospitals in Italy’s Veneto region for unstable angina, non-ST elevation myocardial infarction (NSTEMI), or ST-elevation myocardial infarction (STEMI). The aims of the ABC Study were (1) long-term follow-up with regard to both fatal and non-fatal events, and (2) evaluation of the prognostic value of several clinical variables at baseline. The criteria for a diagnosis of ACS are based on the clinical presentation and electrocardiography, as well as the presence of biochemical markers of necrosis in the patient’s serum (15, 16).

**Figure 1:**
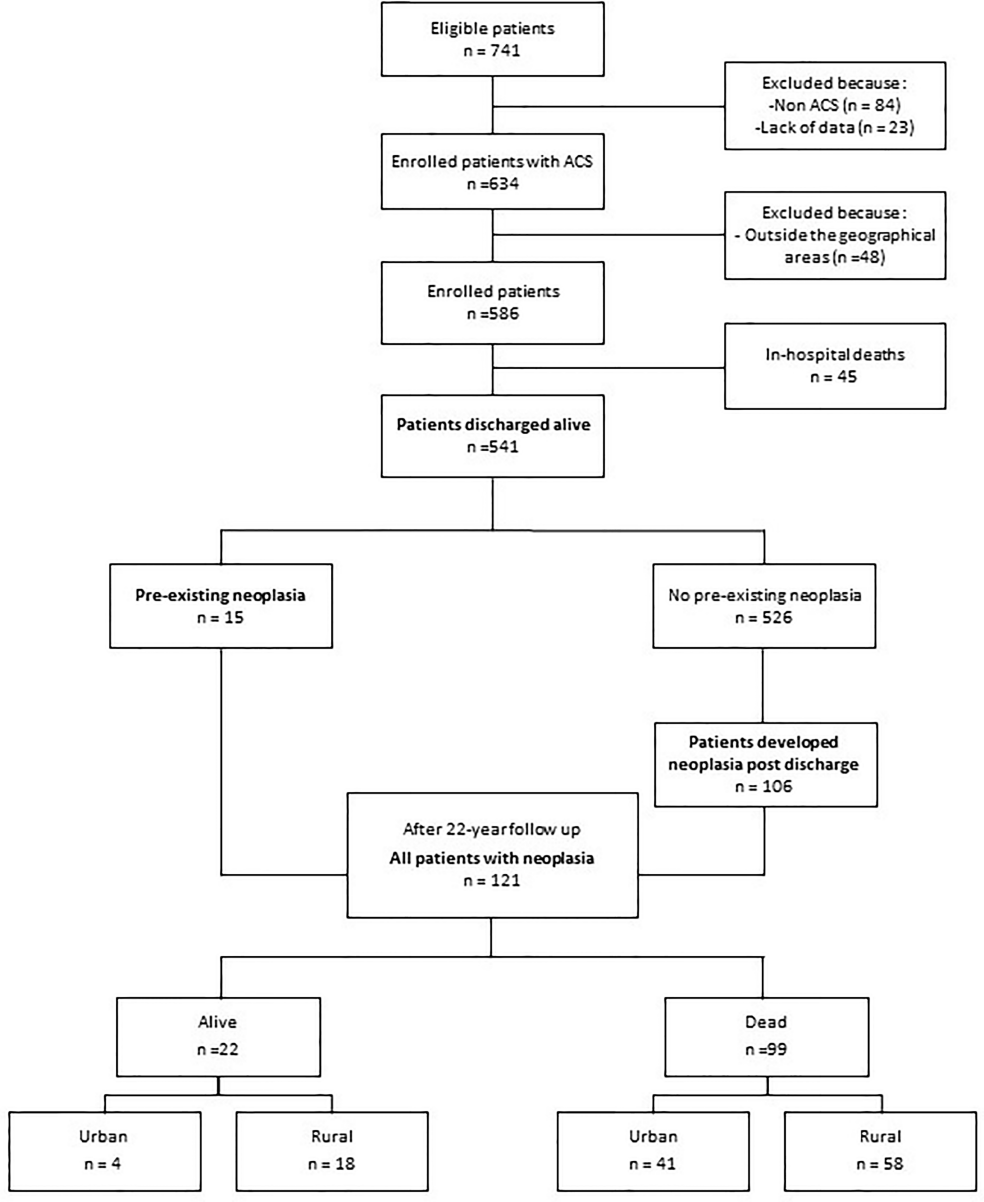
Flow diagram of the study population and progress during follow-up. *ACS, acute coronary syndrome*.

Of the 741 patients considered eligible at the time of admission, the study excluded 84 for having other diseases, 23 due to missing baseline data, and 48 due to their residing outside the Veneto region. Forty-five of the remaining 586 patients died during the index hospitalization, leaving 541 patients in the post-discharge follow-up (**Figure 1**). Data were anonymised through codes, and the study data did not include personal data or identifiers. Written informed consent was obtained from all enrolled patients. The protocols were approved by the ethics committees at the participating hospitals and were carried out in accordance with the Declaration of Helsinki.

### Urban-Rural Classification

As previously described in detail (14), the ABC study includes patients admitted to hospitals in the following cities in Italy’s Veneto region: Conegliano-Vittorio Veneto in Treviso province (northern), Bassano in Vicenza province (central), and Adria-Cavarzere in Rovigo province (southern). Residency was categorized as urban or rural in each province (six geographic areas) using the Rural Development Programme (Programma di Sviluppo Rurale [PSR] 2014-2020) classification of municipalities, which categorises municipalities based on their rural or urban nature (17). The entire study area has a total population of 586,976 that is 24% urban and 76% rural (17, 18).

### Measurements and Follow-Up

A thorough medical history was collected from the patient’s medical records, as well as patient interviews, at the time of enrolment. Clinical and laboratory data to be analysed at baseline were obtained during the first week of the patient’s hospitalization. The diagnosis of ACS was based on the presence of at least two of the following criteria: typical changes in serum enzymes (e.g., total creatine kinase and creatine kinase MB), typical electrocardiogram changes (i.e., localized ST-T changes and/or pathological Q waves in at least two contiguous leads), and central chest pain lasting more than 30 minutes (19). The measured variables have been described in detail elsewhere (15, 16).

Clinical check-ups were given to each patient 1, 3, 5, 7, 10, 12, 15, 17, 20, and 22 years after recruitment. Two cardiologists at each hospital monitored the patient cohort throughout the follow-up period. The sources of data were the public health-care administration, family doctors, hospital records, scheduled examinations, medication records from the index hospitalization and follow-up visits, post-mortem examinations, and death certificates. Neoplastic disease present at the index admission, the first clinically documented diagnosis of new malignancy, and causes of death were recorded. All post-enrolment data were recorded prospectively according to the ABC Study on Heart Disease protocol (15). Two different datasheets were used to record baseline and follow-up data, which were merged after the follow-up was completed.

### Statistical Analysis

Data were analysed as continuous variables or proportions. When appropriate, positively skewed distributions were corrected by applying log transformations. We analysed categorical variables using Pearson’s chi-squared and used the unpaired Student’s t-test for measured variables. The data of patients who dropped out before the end of the follow-up period were censored at the time.

To test the homogeneity of the risk of malignancy among the geographic areas evaluated in this study, we used the Breslow-Day test and set p<0.05 to indicate dis-homogeneity of the odds ratios (ORs).

We estimated the risk of neoplastic death using non-adjusted and adjusted Cox regression and Fine-Gray competing risk regression models. We assessed a formal interaction term for neoplastic mortality risk between geographic areas in all models to study effect modification. We used Schoenfeld residuals with 95% confidence intervals (CIs) to test the proportionality assumption and quantified the risk estimates as hazard ratios (HRs) and sub-hazards ratios (SHRs). The following variables were included in all fully adjusted models: age, sex, education level, presence of heart failure at admission, baseline serum cholesterol, smoking, alcohol consumption, and anti-platelet and beta-blockers time-intensity treatment per cent during follow-up.

Marginal post-estimation analysis was used to graphically show the predicted relative hazards and sub-hazards of death due to malignancy across the northern, central, and southern provinces.

Categorical variables were summarized as numbers and percentages and continuous variables as the medians and interquartile ranges. Two-tailed p<0.05 was considered significant. The software STATA 14 (College Station, Texas, USA) was used for all statistical analyses.

## Results

### Study Population and Baseline Characteristics

The 541 patients enrolled in the three provinces included 249 (46%) residing in urban areas and 292 (54%) in rural areas. The two groups shared most of their demographic and baseline clinical characteristics (**Table 1**). Follow-up was completed by all surviving patients (representing 6232 person-years, which indicates the total analysis time at risk of all patients under observation), with the exception of three patients who were censored when they withdrew consent (n=2) or moved overseas (n=1).

**Table 1:** Demographic and clinical characteristics of patients with ACS who were discharged alive, according to the geographic area of residency.

Variable	Overall sample (n = 541)	Urban areas (n = 249–46%)	Rural areas (n = 292–54%)	*p*-value
Age in years	67 (58–74)	67 (58–76)	66 (58–74)	0.21
Female gender	30%	30%	29%	0.95
Education above primary school	25%	32%	18%	0.001
Body mass index, kg/m^2^	25.7 (23.9–28.1)	25.6 (23.6–27.7)	25.7 (24.2–28.4)	0.05
Smoking habit^*^	67%	67%	66%	0.81
Alcohol use	75%	75%	74%	0.83
Hypertension	48%	51%	46%	0.20
Diabetes mellitus	23%	25%	21%	0.19
Systolic blood pressure, mmHg	120 (110–130)	120 (110–130)	120 (110–135)	0.66
Diastolic blood pressure, mmHg	80 (70–80)	75 (70–80)	80 (70–80)	0.32
Heart rate, beats/min	70 (60–82)	72 (60–80)	70 (60–82)	0.83
Non-ST elevation ACS	38%	37%	40%	0.40
Killip class >1	33%	37%	30%	0.08
Hb, g/dL	14 (12–15)	14 (12–15)	14 (13–15)	0.86
Blood glucose level, mg/dL	120 (100–158)	125 (103–167)	117 (99–151)	0.16
Serum creatinine level, mg/dL	0.9 (0.98–1.1)	0.9 (0.8–1.1)	0.9 (0.9–1.1)	0.64
CK-MB peak^†^, U/L	102 (42–203)	104 (44–212)	98 (40–200)	0.58
Total cholesterol^†^, mg/dL	207 (178–243)	205 (175–237)	209 (179–243)	0.28
Treatment during follow-up ^‡^				
Anti-platelet, %	87%	88%	87%	0.93
B-Blockers, %	54%	52%	56%	0.35

The values are presented as median (interquartile range) or percentages.

ACS, acute coronary syndrome; CK-MB, creatine kinase-MB isoenzyme; Hb, haemoglobin.

^*^Previous smokers and currently smoking patients. ^†^P-values were calculated using log-transformed data. ^‡^Treatment received at any time during follow-up.

### Cancer and Malignancy Death Risk by Geographic Area

A total of 121 patients had malignancy, which was pre-existing at enrolment in 15 patients and developed during follow-up in 106 (**Figure 1**). The most common sites were the lungs (22%), colorectal (19%), and prostate (15%), as well as the pancreas (5%), breast (5%), and leukaemia (5%). A total of 99 (18%) patients died due to malignancy (**Figure 1**). The risk of cancer death was associated with important risk factors, such as age and smoking. However, an inverse association between cancer death risk and serum cholesterol was found (HR=0.49; 95% CI=0.32-0.74; p= 0.001).

**Figure 2:**
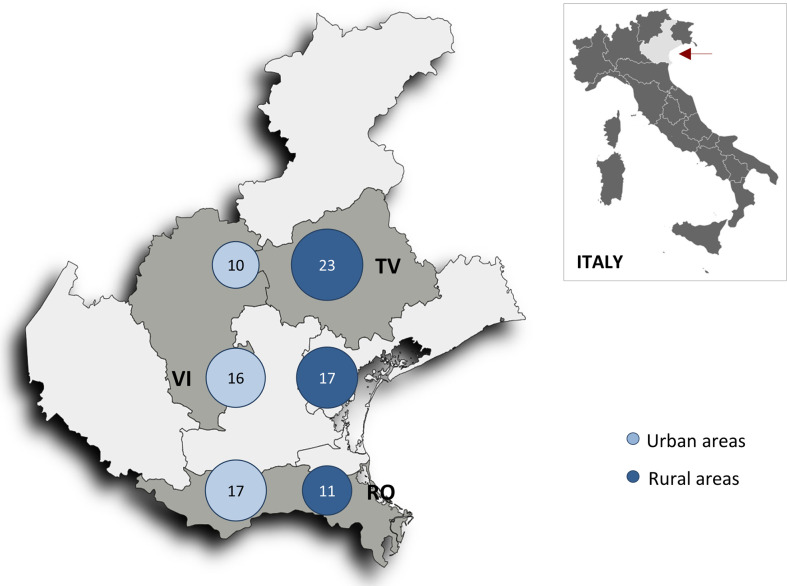
Map of the Veneto region (red arrow) showing neoplastic death rates per 1000 person-years in the six geographic areas (n = 541 patients). *RV, Rovigo province; TV, Treviso province; VI, Vicenza province*.

The overall incidence rate of death from cancer was 16/1000 person-years; it was slightly higher in rural areas compared to urban areas (17 and 15/1000 person-years, respectively), with the highest incidence observed in the northern rural area (**Figure 2**).

Differences in cancer death risk among the six geographic areas were examined using Breslow-Day test of homogeneity comparing urban and rural areas risk, and the risk passing from southern to northern provinces. Significant differences in cancer death risk going from urban and rural areas (OR = 2.9; 95%Cl= 1.2-8.1 in the north, 1.4: 95%Cl= 0.5-4.2 in the central, and 0.7; 95%Cl= 0.3-1.5 in the southern province; *p* = 0.03), and going from south to north (OR = 0.6; 95%Cl= 0.3-1.2 in urban, and 1.9; 95%Cl= 0.9-3.9 rural areas; *p* = 0.01), were revealed by the Breslow-Day test of homogeneity. As for non-neoplastic death risk, no significant difference between the urban and rural areas (OR = 0.6; 95%Cl= 0.3-1.1 in the north, 0.5; 95%Cl= 0.2-1.2 in the central, and 0.9; 95%Cl= 0.5-1.7 in the southern province; *p* = 0.35) or from the southern to northern province (OR = 1.7 in urban, and 1.0 in rural areas; *p* = 0.14) was detected using the same homogeneity test.

The unadjusted Cox regression analysis did not reveal a significant change in HR for neoplastic death between the urban and rural areas, or between the southern and northern provinces (**Table 2**). However, inclusion of an interaction term yielded a strong positive interaction, with the risk increasing between urban and rural areas from the southern to the central to the northern province.

**Table 2:** Cox regression and Fine-Gray competing risk regression analysis of non-neoplastic and neoplastic mortality risk over 22 years of follow-up after ACS with the interaction for risks between the six geographic areas (n = 541).

Variable	Unadjusted			Fully adjusted^*^		
	HR (95% CI)	Z	*p*	HR (95% CI)	Z	*p*
**Cox regression**						
**Non-neoplastic mortality (n = 321)**						
urban-rural areas	0.8 (0.7–1.0)	-1.8	0.08	0.9 (0.7–1.1)	-1.2	0.24
southern-northern provinces	1.1 (1.0–1.3)	1.6	0.10	1.0 (0.8–1.1)	-0.4	0.71
Interaction (urban/rural areas and south to north provinces)	1.0 (0.7–1.2)	-0.3	0.74	1.1 (0.9–1.4)	0.8	0.41
**Neoplastic mortality (n = 99)**						
urban-rural areas	1.1 (0.7–1.7)	0.5	0.59	1.3 (0.8–1.9)	1.1	0.27
southern-northern provinces	1.1 (0.9–1.4)	1.1	0.29	1.0 (0.8–1.3)	0.3	0.74
Interaction (urban/rural areas and south to north provinces)	1.9 (1.1–3.0)	2.4	0.01	2.1 (1.3–3.4)	2.9	0.003
**Fine-Gray competing risk regression analysis**						
	**SHR (95% CI)**	**Z**	***p***	**SHR (95% CI)**	**Z**	***p***
**Non-neoplastic mortality (n = 321)**						
urban-rural areas	0.8 (0.7–1.0)	-1.8	0.07	0.8 (0.7–1.1)	-1.4	0.17
southern-northern provinces	1.1 (1.0–1.2)	1.2	0.25	1.0 (0.8–1.1)	-0.2	0.82
Interaction (urban/rural areas and south to north provinces)	0.9 (0.7–1.1)	-0.9	0.38	0.9 (0.7–1.2)	-0.5	0.60
**Neoplastic mortality (n = 99)**						
urban-rural areas	1.2 (0.8–1.8)	0.9	0.32	1.3 (0.8–1.9)	1.2	0.25
southern-northern provinces	1.1 (0.8–1.3)	0.5	0.60	1.1 (0.9–1.4)	0.8	0.45
Interaction (urban/rural areas and south to north provinces)	1.8 (1.1–3.0)	2.4	0.01	1.9 (1.1–3.0)	2.6	0.01

CI, confidence interval; HR, hazard ratio; SHR, sub-hazard ratio.

P-values were calculated for log-transformed data. *Adjusted for age, sex, smoking, education level, alcohol consumption, baseline serum cholesterol, presence of heart failure at admission, and anti-platelet and beta-blockers time-intensity treatment per cent during follow-up.

**Figure 3:**
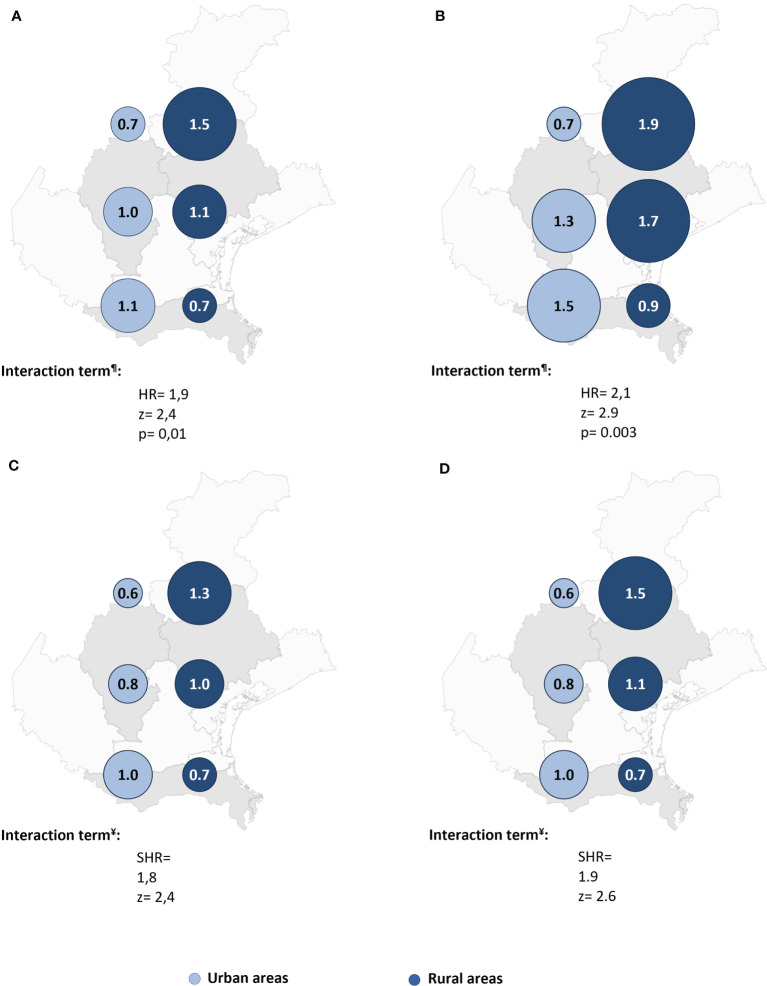
The predicted relative hazards and sub-hazards of neoplastic mortality 22 years after acute coronary syndrome in the six geographic areas (n = 541 patients). HR, Hazard ratio. SHR, Sub-hazards ratio. The relative hazards were calculated using margins post estimation of the unadjusted **(A)**, and the fully adjusted **(B)** Cox regression analysis. The relative sub-hazards were calculated using margins post estimation of the unadjusted **(C)**, and the fully adjusted **(D)** Fine-Gray competing risk regression analysis. ^¶^Calculated using Cox regression analysis. ^¥^Calculated using Fine-Gray competing risk regression analysis.

Cox regression post-estimation marginal analysis indicated that the predicted relative hazard of death due to malignancy was highest in the northern rural areas (**Figure 3A**). This result held true even with full adjustment of the Cox regression model (**Table 2** and **Figure 3B**), and the unadjusted and fully adjusted Fine-Gray competing risk regression models yielded similar results (**Table 2**). Margins post estimation of the unadjusted (**Figure 3C**), and the fully adjusted (**Figure 3D**) Fine-Gray competing regression analysis showed the highest predicted relative sub-hazard of cancer in the northern rural areas.

**Figure 4:**
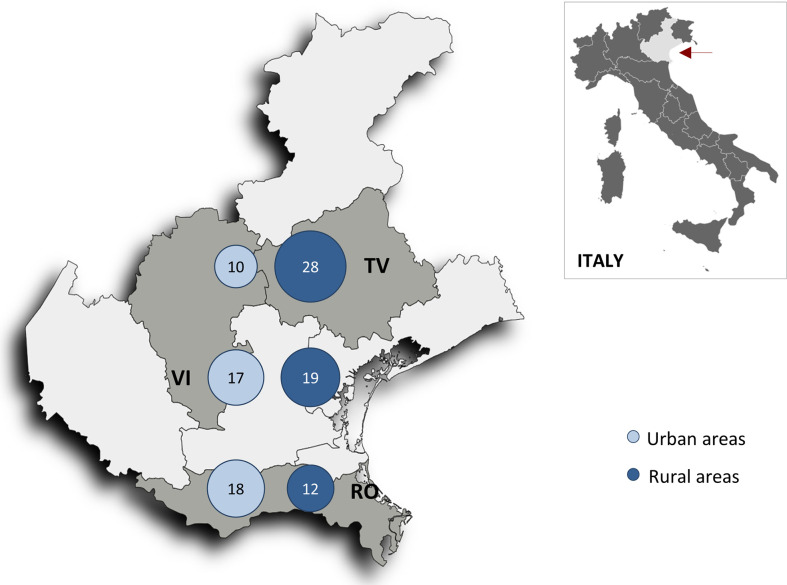
Map of the Veneto region (red arrow) showing neoplastic death rates per 1000 person-years in the six geographic areas after excluding patients who had malignancy and still alive (n = 519 patients). *RV, Rovigo province; TV, Treviso province; VI, Vicenza province*.

**Figure 5:**
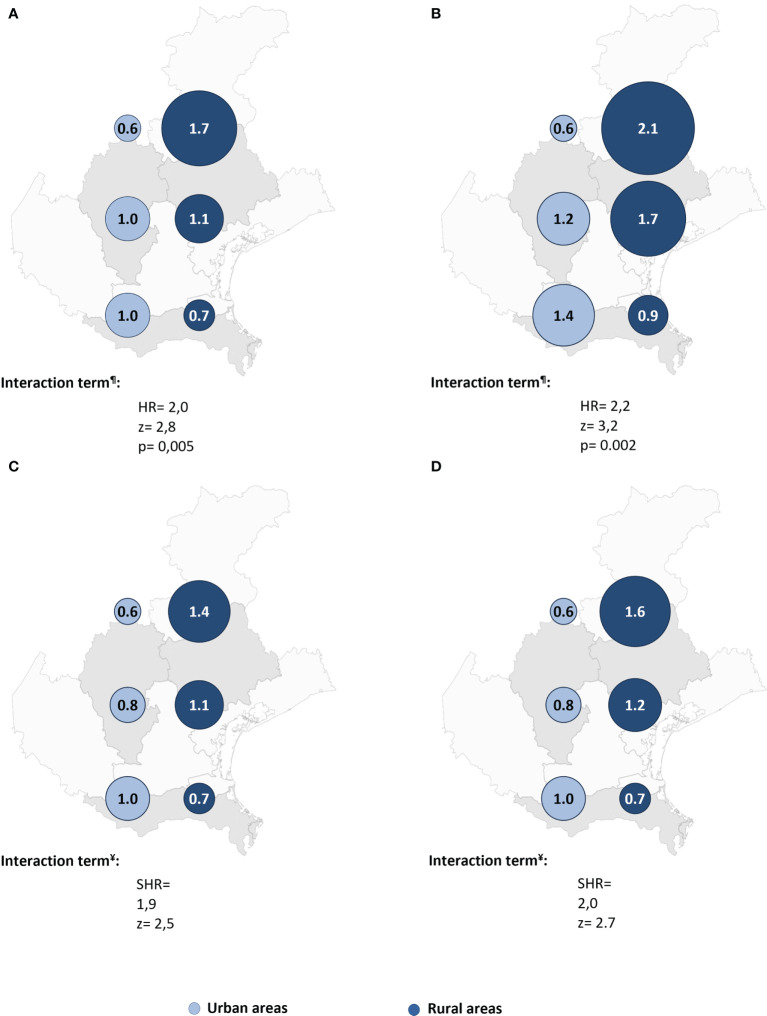
The predicted relative hazards and sub-hazards of neoplastic mortality 22 years after acute coronary syndrome in the six geographic areas after excluding patients who had malignancy and still alive (n = 519 patients). SHR, Sub-hazards ratio. The relative hazards were calculated margins post estimation of the unadjusted **(A)**, and the fully adjusted **(B)** Cox regression analysis. The relative sub-hazards were calculated using margins post estimation of the unadjusted **(C)**, and the fully adjusted **(D)** Fine-Gray competing risk regression analysis. ^¶^Calculated using Cox regression analysis. ^¥^Calculated using Fine-Gray competing risk regression analysis.

Finally, to avoid the influence of alive-neoplastic patients on surviving analysis, we ran the same analyses, after exclusion of the 22 patients who developed malignancy but survived to the end of follow-up. Interestingly, these results were virtually the same, and even a bit stronger (**Table 3** and **Figures 4**, **5**).

**Table 3:** Cox regression and Fine-Gray competing risk regression analysis of non-neoplastic and neoplastic mortality risk over 22 years of follow-up after ACS with the interaction for risks between the six geographic areas after excluding patients who had malignancy and were still alive (n = 519).

Variable	Unadjusted			Fully adjusted^*^		
	HR (95% CI)	Z	*p*	HR (95% CI)	Z	*p*
**Cox regression**						
**Non-neoplastic mortality (n = 321)**						
urban-rural areas	0.9 (0.7–1.0)	-0.9	0.33	0.9 (0.7–1.1)	-0.8	0.43
southern-northern provinces	1.2 (1.0–1.3)	2.3	0.02	1.0 (0.9–1.2)	0.1	0.95
Interaction (urban/rural areas and south to north provinces)	1.0 (0.8–1.3)	0.2	0.82	1.2 (0.9–1.5)	1.1	0.28
**Neoplastic mortality (n = 99)**						
urban-rural areas	1.2 (0.8–1.8)	1.0	0.30	1.3 (0.9–2.0)	1.4	0.15
southern-northern provinces	1.2 (0.9–1.5)	1.5	0.13	1.1 (0.9–1.4)	0.7	0.46
Interaction (urban/rural areas and south to north provinces)	2.0 (1.2–3.3)	2.8	0.005	2.2 (1.4–3.6)	3.2	0.002
**Fine-Gray competing risk regression analysis**						
	**SHR (95% CI)**	**Z**	***p***	**SHR (95% CI)**	**Z**	***p***
**Non-neoplastic mortality (n = 321)**						
urban-rural areas	0.9 (0.7–1.1)	-1.2	0.23	0.9 (0.7–1.1)	-1.1	0.28
southern-northern provinces	1.1 (1.0–1.3)	1.6	0.10	1.0 (0.9–1.2)	0.03	0.97
Interaction (urban/rural areas and southern to northern provinces)	0.9 (0.7–1.2)	-0.5	0.60	0.9 (0.7–1.2)	-0.4	0.70
**Neoplastic mortality (n = 99)**						
urban-rural areas	1.3 (0.9–1.9)	1.2	0.21	1.3 (0.9–1.9)	1.4	0.17
southern-northern provinces	1.1 (0.9–1.4)	0.7	0.49	1.1 (0.9–1.4)	1.0	0.31
Interaction (urban/rural areas and southern to northern provinces)	1.9 (1.2–3.1)	2.5	0.01	2.0 (1.2–3.4)	2.7	0.007

CI, confidence interval; HR, hazard ratio; SHR, sub-hazard ratio.

P-values were calculated for log-transformed data. *Adjusted for age, sex, smoking, education level, alcohol consumption, baseline serum cholesterol, presence of heart failure at admission, and anti-platelet and beta-blockers time intensity treatment per cent during follow-up.

Neither an urban/rural difference in non-neoplastic death risk nor a significant interaction between the six geographic areas was observed (**Table 2**).

## Discussion

In this study, we analysed urban-rural disparity in cancer death risk long after ACS in the Veneto region, Italy. Our main result is a significant and independent difference in long-term risk of cancer death among urban and rural areas. This result supports geography as a strong independent effect modifier of the risk of cancer death in ACS patients. For patients in the northern province who survived ACS, living in a rural area as opposed to an urban area increased the probability of cancer death while in the southern province, living in a rural area decreased the probability of cancer death.

We recently reported that ACS patients have a higher long-term risk of cancer than the general population (4). Among lifelong ACS patients, we also documented a significant difference in the incidence and prevalence of malignancy across different parts of the Veneto region, with the northern rural area having the greatest risk (14).

To our knowledge, this study is the first to report a geographic difference in cancer death risk in ACS with a long follow-up (22 years) and virtually no dropouts.

In line with current knowledge, we found that cancer death risk is associated with important risk factors, such as age and smoking, and found an inverse association between cancer death risk and serum cholesterol in rural and urband areas concordant to previous reports (20–22).

Several other studies have reported increased cancer death risk in the general populations of rural areas versus urban areas (23–27). Researchers have analysed urban-rural variations in cancer incidence and mortality among general populations for many years and have attributed that variation to the lifestyle dissimilarities between these different geographic areas, such as socioeconomic status, smoking, diet, and differing exposures to other risk factors (24–26, 28).

Nevertheless, in our specific cohort of ACS patients, we cannot yet explain the approximately 2.5-fold higher cancer death risk in the northern rural area compared with that in the northern urban area. Most of the clinical characterstics of our patients from urban and rural areas were similar.

Urban-rural disparities in cancer survival are strongly influenced by socioeconomic factors. The rural populations had less higher education (**Table 1**) and, in the general population, education level is reported to be inversely related to cancer death risk (28, 29). However, the higher risk of malignancy in the northern rural area was not affected by adjustment for education level.

The patients in our study were living in the same region, with no major differences in their exposure to common risk factors for cancer. Homogeneous exposure across urban and rural areas has also been reported recently, particularly in Western European countries, where it can be explained by rapid economic development, an increasingly shared lifestyle, and more opportunity for relocation (25–27).

The geographic difference in cancer death risk might also reflect a different distribution of the health services offered. In more disadvantaged regions, cancer survival is reported to be lower, even after adjusting for the stage at diagnosis (30–33). Yet, the six areas included in this study are served by the same primary health-care services, with no more than 1500 patients cared for by any general practitioner. All residents are covered by mandatory national health insurance, which provides free primary and hospital care. A new comprehensive primary care model was recently adopted in the Veneto Region in which at least four general practitioners work with nurses, specialists, social workers, and other health professionals (34, 35). Thus, it seems unlikely that the differences in cancer death risk reported here can be ascribed to health system disparities.

The higher cancer mortality in residents of the northern rural area in our study may be caused by differences in cancer incidence, as we have previously reported a higher incidence of cancer in the same area (14). Jansen et al. have also attributed the higher cancer mortality in the more deprived areas in their study to a disparity in cancer incidence (36).

A main strength of our study is the long follow-up of an unselected sample of ACS patients, with almost no dropouts. To our knowledge, it is the first to report such a difference in cancer death risk in a specific population between urban and rural areas. Although, our study has limitations, one limitation is that patients were enrolled before percutaneous coronary angioplasty was used to treat STEMI, so early mechanical reperfusion may have altered the results. A second limitation is that the diagnoses of myocardial infarction pre-dated the use of troponin as a marker of necrosis. Instead, we relied on altered creatine kinase and creatine kinase-MB levels, which are still recommended for use when troponin data are unavailable (37). Another limitation is the lack of data about certain individual and environmental risk factors related to cancer development and mortality. Nevertheless, most major risk factors were recorded and included in the fully adjusted models. Even though the present analysis was carried out on a relatively small sample of patients, the results appear to be consistent and coherent across the very long term of follow-up (22years). We also acknowledge the preliminary nature of these results. Nevertheless, they highlight the influence of territory on such a severe disease like malignancy with subsequent cancer death not only in the general population but in post ACS patients specifically. These preliminary results aim to draw (even more) our attention to the importance of cancer prevention even through environmental care. Finally, the patients in our cohort were all Caucasian, and our conclusions cannot be generalized to other regions, populations, or ethnic groups.

This prospective study of unselected, real-world ACS patients showed that cancer death risk differs significantly in different parts of the Veneto region of Italy, with the highest risk in the northern rural area. This study emphasizes the importance of investigating the aetiological factors related to higher cancer death risk in particular areas and of implementing a preventive policy based on area-specific knowledge.

## Data Availability Statement

The raw data supporting the conclusions of this article will be made available by the authors, without undue reservation.

## Author Contributions

GB and HM designed the study. RC, and RP contributed to the original data collection. FC and RC contributed to data handling and patient follow-up. GB and HM contributed to the data analysis and interpretation, tables, figures, and manuscript preparation. MA-W and TN contributed to statistics and figure revision. All authors contributed to ensuring the accuracy of the data analysis. All authors contributed to the article and approved the submitted version.

## Funding

This work was supported by a grant from the Veneto region in Italy (Veneto Region Act no. 748, Venice, May 14, 2015, grant number 2987929). University of Padova (Padova, Italy) contributed to and supported data collection, management, and analysis. The ABC Study on Heart Disease Foundation-ONLUS provided intellectual support for the present study.

## Conflict of Interest

The authors declare that the research was conducted in the absence of any commercial or financial relationships that could be construed as a potential conflict of interest.

## Publisher’s Note

All claims expressed in this article are solely those of the authors and do not necessarily represent those of their affiliated organizations, or those of the publisher, the editors and the reviewers. Any product that may be evaluated in this article, or claim that may be made by its manufacturer, is not guaranteed or endorsed by the publisher.
